# Kisspeptins inhibit ectopic endometrial cell invasion and angiogenesis by suppressing PI3K/AKT signaling pathway via CREB5 in endometriosis

**DOI:** 10.7150/ijms.112890

**Published:** 2026-01-01

**Authors:** Lingnan Kong, Suliya Yushanjiang, Li Nie, Yiran Pan, Lei Jin, Zun Wang, Man He, Gao Zhang, Ziyang Ma, Rongqian Zhao, Dongzhi Yuan, Changlong Li

**Affiliations:** 1 West China School of Basic Medical Sciences & Forensic Medicine, Sichuan University, Chengdu, China.; 2 Department of Public Health Laboratory Sciences, West China School of Public Health and West China Fourth Hospital, Sichuan University, Chengdu, China.

**Keywords:** endometriosis, KISS1, PI3K/AKT signaling pathway, cell invasion, angiogenesis

## Abstract

Endometriosis (EMs) is a common gynecological disorder. According to the most widely recognized theory of retrograde menstruation, endometrial cells require completion of three key steps during ectopic implantation: adhesion, invasion, and angiogenesis. Although kisspeptin exerts anti-invasive and anti-angiogenic effects in multiple tumors, its potential inhibitory effects mediated through the KISS1 receptor (KISS1R) on EMs-related invasion and angiogenesis remain uncharacterized. This study aimed to identify regulatory genes in EMs pathogenesis via RNA sequencing and elucidate underlying molecular mechanisms. We performed a comparative transcriptomic analysis of ectopic endometrium (EC) and eutopic endometrium (EU) in 5 patients with EMs and control endometrium in 3 women without EMs. Then, we screened for genes that showed significant differences between EU and group, and even more pronounced differences between EC and EU, indicating a progressive change. Moreover, we identified the top 20 progressively altered genes during EMs development, including KISS1R. Furthermore, KEGG pathway analysis revealed that these progressively altered DEGs were mainly enriched in the phosphatidylinositol 3-kinase (PI3K)/ protein kinase B (AKT) signaling pathway. Previous studies suggest that the PI3K/AKT signaling pathway may mediate cell invasion and angiogenesis in EMs. Additionally, substantial evidence indicates that cAMP-response element binding protein (CREB5), a transcriptional regulator, can regulate the PI3K/AKT pathway. However, the mechanism of functional changes of CREB5 and PI3K/AKT in EMs is unclear. Our study validated the functional role of KISS1/KISS1R in EMs through animal experiments and cell experiments. It may suppress the cell invasion and angiogenesis of endometrial cells by reducing the phosphorylation levels of PI3K and AKT mediated by increasing CREB5. This mechanistic insight provides novel pathogenic explanations for EMs.

## 1. Introduction

Endometriosis (EMs) is a common gynecological disorder characterized by ectopic implantation of active endometrial cells 1. It affects approximately 10% of women of childbearing age, causing dysmenorrhea, chronic pelvic pain and infertility 2. Epidemiological data reveal that the incidence of EMs is increasing annually, with a higher prevalence in Asian populations compared to European populations 3. Despite its benign histological appearance, it exhibits tumor-like clinical behavior, including invasiveness and metastasis. In moderate to severe cases, the extensive lesions can severely impact patients' quality of life 4. Currently, the medical community has yet to reach a definitive conclusion regarding the specific origin and pathogenesis of EMs, which has contributed to the challenges in diagnosis and treatment 5. So far, the theory of retrograde menstruation is the most broadly accepted hypothesis for potential pathogenesis of EMs, but this theory does not fully explain why only a small number of women with common retrograde menstruation develop EMs. Based on the theory of retrograde menstruation, endometrial cells need to undergo at least three steps of adhesion, invasion and angiogenesis to achieve ectopic implantation and growth. To complete this process, alterations in the biological characteristics of the eutopic endometrium (EU) are required, enhancing its adhesion, invasion, and angiogenic capabilities. Subsequently, some scholars discovered that there were differences in the expression of adhesion, invasion and angiogenesis related molecules in the EU of both patients with EMs and normal women 6, 7. Therefore, the theory of endometrial determinism was further proposed to supplement the theory of retrograde menstruation. Consistent with the above theory, cell invasion and angiogenesis are considered to be key steps in successful implantation of ectopic endometrium (EC). Despite extensive investigation, their molecular regulators remain incompletely characterized.

Kisspeptins, encoded by the KISS1 gene, were initially identified as metastasis-suppressing factors in human melanoma by Lee *et al.* in 1996 8. Thereafter, KISS1 gene was cloned and located on human chromosome 1q32-1q41, with a total length of 771 bp, and its gene structure comprises 4 exons and 3 introns. Kisspeptins are a family of 145-amino acid-containing neuropeptides that are hydrolyzed by Flynn protease into Kisspeptin-54, -14, -13, and -10 with equal affinity and biological activity 9. These kisspeptins are highly conserved in mammals, and human kisspeptin-10 (KP10) differs from mouse KP10 by only one amino acid. Numerous studies have demonstrated that the four short peptides of kisspeptins can bind to their receptor KISS1 receptor (KISS1R), and play the same biological function 10. Kisspeptins have been found to be involved in a variety of biological functions, including controlling the onset of puberty, anti-tumor cell invasion, regulating the hypothalamic-pituitary-gonadal axis, and maintaining the balance of glucose and lipid metabolism 11, 12. Moreover, studies have proved that kisspeptins exhibit anti-angiogenesis and anti-invasion activity in many malignant tumors, including pancreatic cancer, colorectal cancer and ovarian cancer 13-15. Over the past few decades, the relevant research on the relationship between KISS1 and EMs has been remarkably limited. Importantly, whether kisspeptins can inhibit invasion and angiogenesis of EC through KISS1R has also not been reported.

Recent studies have demonstrated that kisspeptin-10 inhibits MMP9 and VEGF expression in cerebral aneurysm mice, suppressing angiogenesis 16. Similarly, upregulation of KISS1 in renal cancer cells activates the PI3K/AKT pathway and significantly reduces their invasive capacity 17. In recent years, growing evidence indicates PI3K/AKT pathway activation in EMs. Daniela's research group observed not only upregulated PI3K protein expression but also enhanced AKT phosphorylation in EMs lesions 18. The Arisa's team demonstrated significantly elevated AKT phosphorylation levels in both human oocyte EMs patients and mouse EMs models compared to non- EMs controls 19. These findings suggest that KISS1/KISS1R may regulate cell invasion and angiogenesis through the PI3K/AKT signaling pathway, although the precise molecular mechanisms require further investigation. Additionally, while CREB5 has been identified as a transcriptional regulator of the PI3K/AKT signaling pathway 20, its potential role in EMs remains uninvestigated.

In this study, we analyzed relevant pathways involved in the progression of EMs and identified candidate biomarkers. First, we screened for differentially expressed genes (DEGs) among the EU and EC of EMs patients, as well as the control endometrium from non-EMs individuals. Our results revealed that KISS1/KISS1R exhibited a progressive change across the three groups, suggesting a potentially significant role in the development of EMs. Then, enrichment analyses for DEGs were performed, focusing on phosphatidylinositol 3-kinase (PI3K)/ protein kinase B (AKT) signaling pathway. Next, the function of KISS1/KISS1R was further verified in animal and cell experiments. We established both autologous and allogeneic transplantation models to validate our findings in animal experiments. In cell experiments, we used scratch assay, transwell invasion assay, and tube formation assay, along with CREB5 knockdown, to investigate the molecular mechanisms by which KISS1 blocks EC cell invasion and angiogenesis through KISS1R. Numerous previous studies have demonstrated that CREB5, as a recognized transcription factor, is an upstream molecule of the PI3K/AKT signaling pathway. Our data suggest that KISS1 inhibits cell invasion and angiogenesis by blocking CREB5-mediated PI3K/AKT signaling pathway activation.

## 2. Material and Methods

### 2.1. Patients and clinical samples

Patients with ovarian EMs were diagnosed according to typical clinical symptoms of dysmenorrhea and imaging reports of transvaginal ultrasound. All samples were collected from West China Second Hospital of Sichuan University. All participants in this study were Han Chinese, Asian population.

In this research, five patients with ovarian EMs and three women with no EMs as matched controls were included. All participants recruited in our study were women of reproductive age with normal menstrual cycles (26-32 days). The enrolled patients had not received hormone therapy for at least 3 months before surgery. All EC samples in our study were collected through laparoscopic ovarian cystectomy, and the matched EU samples were obtained via uterine curettage. The endometrium samples in the control group were also collected by uterine curettage from women with benign conditions other than EMs, such as fibroids and endometrial polyp, and were laparoscopically confirmed to have no EMs.

This study was approved by the Human Research Ethics Committee of Sichuan University (No. 2021012), and informed consent was obtained from all the participants.

### 2.2. RNA-sequencing and data analysis

According to manufacturer's instructions, total RNA was extracted from EC and EU samples of EMs patients, as well as from the control endometrium of non-EMs individuals, using RNA sample Total RNA Kit (DP419, TIANGEN). The mRNA-seq Library was prepared using U-mRNAseq Library Prep Kit (AT4221, KAITAI-BIO) with Ribo-off rRNA Depletion Kit (Bacteria) (N407, Vazyme). Libraries were pooled and sequenced using the Illumina NovaSeq machine as 150-bp paired-end sequencing reads. Clean reads were mapped to the reference genome using HISAT2 (v2.2.1,http://daehwankimlab.github.io/hisat2/). The DEGs were identified by edgeR (https://bioconductor.org/packages/release/bioc/html/edgeR.html), and genes with FDR <0.05 and |log2 foldchange| > 1 were identified as DEGs. Kyoto Encyclopedia of Genes and Genomes (KEGG) pathway enrichment analyses were performed to show the biological functions of differentially expressed mRNAs.

### 2.3. Quantitative PCR

The remaining RNA after sequencing of each sample was used for quantitative real-time polymerase chain reaction (qPCR). cDNA was synthesized with 5× FastKing-RT SuperMix. The subsequent incubation process was 15 min at 42 °C, then 3 min at 95 °C. The cDNA was stored at 4 °C for further use. Quantitative RT-PCR was conducted using CFX Connect Real-Time PCR Detection system (Bio-Rad, USA). According to the manufacturer's protocol of AceQ qPCR SYBR Green Master Mix (Vazyme, Nanjing, China), the procedure for qPCR was as follows: 95 °C for 5 min to denature the DNA, followed by 95 °C for 10 s and 60 °C with a time of 30 s, and a total of 40 cycles were performed in this step. GAPDH was used as an internal control. All primers were synthesized by Tsingke Biotechnology, Beijing, China, and their sequences are listed in Table 1. In this study, all measurements were repeated at least three times, and the mean of the replication was used for statistical analysis.

### 2.4. Surgical induction of EMs in C57BL/6 female mice

7 weeks old female C57BL/6 mice were purchased from GemPharmatech Co., Ltd. The mice were housed in an environment maintained at a temperature ranging from 20 to 25°C and a humidity ranging from 40 to 50%, with a 12-hour light and 12-hour dark cycle. Before modeling, all mice were given adaptive feeding for 1 week to become accustomed to the experimental conditions. To induce the EMs mouse model, surgical induction was performed using both allogeneic and autologous endometrial transplantation methods. All experimental procedures and animal care are approved by the Ethics Committee of Sichuan University (No. 2021129).

According to the procedure of the surgical induction in allogeneic mice as shown in Figure 2A, this model can simulate the spontaneous development of EMs in women. In brief, the mice were injected with 30 ng estradiol subcutaneously for seven days. After 7 days, the estradiol-treated mice were euthanized and their uteruses were collected in phosphate buffered saline (PBS). The endometrial tissue was carefully cut into small pieces using scissors that are consistent and smaller than 1mm. We then intraperitoneally injected these fragments into the experimental recipient mice using a 1 ml syringe. The mice model of EMs in allogeneic mice was established 14 days later. The ectopic lesions were placed on a scaled measurement paper for sizing and photographing. The presence of enlarged, semi-transparent cystic ectopic lesions with visible vascularization was considered indicative of successful model establishment.

In line with the process of the surgical induction via autologous endometrial transplantation exhibited in Figure 2D, a 2 × 3 mm piece of the right distal uterine horn was minced. Then, this uterine fragment was adhered to the right abdominal wall with 3M Vetbond Tissue Adhesive, with the intimal surface facing the abdominal wall, followed by suturing the peritoneum and skin. The mice model of EMs via autologous endometrial transplantation was established 7 days later.

In both autograft and allograft models, the EU and EC samples were rinsed with sterile PBS and collected in 10% neutral buffered formalin for 24 h, followed by transfer to 70% ethanol for further processing. For intervention, KP10 and its antagonist (kisspeptin-234, KP234) were administered by intraperitoneal injection at a dose of 20 µg per mouse every 72 hours after surgery. The control group received an equivalent volume of normal saline (NS) via the same administration regimen.

### 2.5. Immunohistochemistry

Tissue samples were fixed by 4% formalin for 12-24 hours, dehydrated, embedded in paraffin and cut into 5-µm sections. For staining the sections, the Rabbit two-step detection kit from Beijing Zhongshan Golden Bridge Biotechnology Co., Ltd. was used following the manufacturer's instructions. IHC staining was performed on paraffin sections with antibody against KISS1 (cat. no. DF7133; 1:50; Affinity Biosciences, Inc.), KISS1R (cat. no. ab 100896; 1:200; Abcam), MMP9 (cat. no. ab38898; 1:200; Abcam, Inc.), VEGFA (cat. no. ab 52917; 1:150; Abcam, Inc.), CD31 (cat. no. ab 28364; 1:50; Abcam, Inc.), CREB5 (cat. no. 14196-1-AP; 1:200; Proteintech, Inc.), PPP2R2C (cat. no. 12747-1-AP; 1:20; Proteintech, Inc.), PI3K (cat. no. ab227204; 1:1000; Abcam, Inc.), pPI3K (rabbit polyclonal; 1:1000; cat. no. AF5905; Beyotime, Inc.), AKT (cat. no. #4691; 1:1000; Cell Signaling Technology, Inc.) and pAKT (cat. no. #4060; 1:200; Cell Signaling Technology, Inc.). For semi-quantitative analysis, ten fields were randomly selected from each immunohistochemical section.

### 2.6. Cell culture and KP10 treatment

Human endometrial Ishikawa cells and human umbilical vein endothelial cells (HUVEC), purchased from the Wuhan Pricella Biotechnology Co., Ltd., were cultured in DMEM medium containing 10% fetal bovine serum and 1% Cyano-streptomycin at 37°C and 5% CO_2_. Ishikawa is a highly differentiated endometrial epithelial adenocarcinoma cell widely used in investigations related to EMs 21-23. Intervention of KP10 was performed by adding 0, 1, 10, 100μmol/L KP10 into the culture medium of Ishikawa and HUVEC.

### 2.7. Scratch wound healing assay

Scratch wound healing assays were performed in six-well plates. After observing that the cell concentration reaches 90%, a straight scratch is created with the sterile pipette tip. Three scratches were conducted for each well with three scratch lines. The cells are then incubated to allow migrate to the scratched area. At 24, 48, 72 hours after incubation, the microscope was used to observe and photograph under the same field of view.

### 2.8. Cell invasion assay

A transwell chamber (cat. no. 3428; Corning, Inc.) with an 8µm pore filter was used to detect cell invasion. The upper chamber of the transwell chamber was coated with 40 μL Matrigel and stored at 37°C for 3 h. According to the manufacturer's instructions, 1×10^5^ cells were treated in 200µL serum-free medium and plated in the upper chamber, and the lower chamber was filled with 800µL medium containing 20% fetal bovine serum. After the cells were cultured at 37 °C for 24 hours, the remaining cells in the chamber were gently wiped with a cotton swab. Subsequently, the chamber was placed in 4% paraformaldehyde fix solution, fixed at room temperature for 30 minutes, and stained with crystal violet for 5 minutes. For quantification, the number of transmembrane cells was randomly counted in 5 fields and the average number was calculated.

### 2.9. Tube formation assay

The tube formation assay was conducted according to CORNING® protocol. Briefly, the pre-cooled 96-well plate was coated with Corning ®Matrigel®Matrix 50 µL per well and solidified in the 37°C incubator for 30 minutes. Then, HUVEC was seeded in 96-well plate at a density of 2-5 × 10^5^ cells per well. After every 2 hours, the tubes were photographed with an optical microscope to observe the formation of the tubes.

### 2.10. Transfection

To perform CREB5 knockdown *in vitro* cultured cells, we obtained siRNA targeting the human CREB5 gene from Wuhan Jinkarui Biotechnology Co., Ltd. For the CREB5 knockdown of Ishikawa, 7.5µL CREB5 siRNA (150 pmol) was transfected into the experimental group, while the corresponding negative control siRNA was transfected into the control group. When Ishikawa reached 80% confluence, CREB5 siRNA was transfected into the cells. Following the manufacturer's instructions, Lipofectamine 2000 was used to transfect Ishikawa.

### 2.11. Western blot analysis

The total proteins from cells were extracted in RIPA lysis buffer adding PMSF and quantified using BCA Protein Assay Kit (cat. no. P0012; Beyotime, Inc.). Equal amounts of 50 μg total proteins were prepared and separated by sodium dodecyl sulfate-polyacrylamide gel electrophoresis (SDS-PAGE), electroimprinted onto polyvinylidene difluoride (PVDF) membrane, and then blocked with 5% skim milk at 1 TBST room temperature for 1 hour. The membranes were incubated with the CREB5 (cat. no. 14196-1-AP; 1:1000; Proteintech, Inc.), PPP2R2C (cat. no. 12747-1-AP; 1:1000; Proteintech, Inc.), PI3K (cat. no. ab227204; 1:1000; Abcam, Inc.), pPI3K (cat. no. AF5905; 1:1000; Beyotime, Inc.), AKT (cat. no. 4691; 1:1000; Cell Signaling Technology, Inc.) and pAKT (cat. no. 4061; 1:1000; Cell Signaling Technology, Inc.) primary antibody prepared with Primary Antibody Dilution Buffer (cat. no. P0256; Beyotime, Inc.) at 4°C overnight. Finally, the membranes were incubated with secondary antibody and detected using chemiluminescence reagents (cat. no. 4AW011; 4A Biotech, Inc.) and visualized by the imaging system.

### 2.12. Statistical analysis

The statistical analyses were performed using Prism GraphPad 7.00 software. In the RT-qPCR analyses, the RNA levels of the samples were calculated using the 2^-ΔCt^ method. All of these slides were then analyzed using Image J software. The results of immunohistochemistry, scratch wound healing assay, cell invasion assay, tube formation assay and Western blot were analyzed with ImageJ. The normality was analyzed by Shapiro-Wilk test. When they passed normality test, the 2 groups data with equal variance were analyzed by the independent-sample *t* tests, and those with unequal variance were evaluated by Welch test. When 3 groups data were passed normality and equal variance tests, one-way analysis of variance (ANOVA) was employed. Post hoc test was performed by using Dunnett test. Statistical significance was defined as *p* < 0.05.

## 3. Results

### 3.1 RNA-Seq analysis and identification of DEGs related to the development of EMs

A total of 5 patients with EMs and 3 women without EMs were included in this study. Demographic characteristics of the EMs and control group are shown in Table 2. The revised American Fertility Society (rAFS) classification system, currently the most widely used in clinical practice, is employed for scoring and staging EMs. There was no significant difference in any of the demographic characteristics in both EMs and control groups.

**Figure 1:**
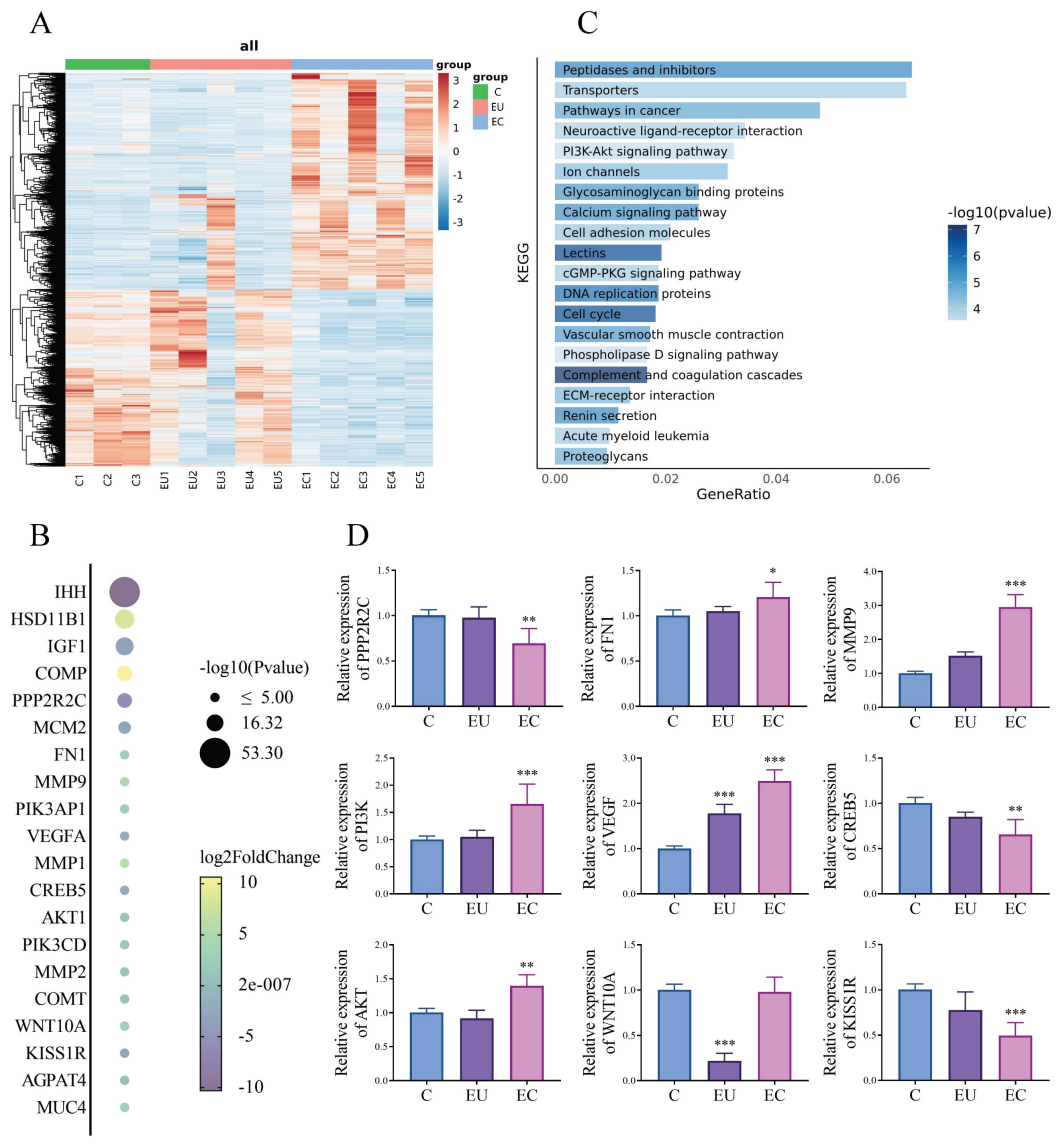
** RNA-Seq analysis and identification of DEGs related to the development of endometriosis (EMs). A:** Hierarchical clustering analysis of genes with differential expression. **B:** The top 20 genes with progressive changes among the differentially expressed genes. **C:** Barplot of enriched Kyoto Encyclopedia of Genes and Genomes (KEGG) pathways among the differentially expressed genes. **D:** The mRNA expression levels of several key genes closely associated with the progressive progression of EMs and the PI3K/AKT signaling pathway in the control (C), eutopic endometrium (EU) and ectopic endometrium (EC) group of clinical samples. n = 3 in C group, n = 5 in both EU and EC group. ^*^p <0.05, ^**^p <0.01, ^***^p <0.001 compared with the C group.

We performed a comparative transcriptomic analysis of EC and EU in 5 patients with EMs and control endometrium in 3 women without EMs. For this study, genes with p < 0.05 and | log2fold changes | >2 were identified as the genes with significant changes. Hierarchical clustering analysis is used to display the DEGs (Fig. 1A). We screened for genes that showed significant differences between the EU group and the control (C) group, and even more pronounced differences between the EC group and the EU group, indicating a progressive change. Then, we have identified the top 20 genes that are likely to show the most progressive changes in the development of EMs among the DEGs (Fig. 1B). For further analysis, KEGG Pathway Analyses were conducted. KEGG pathway analysis revealed multiple enrichment pathways, among which PI3K/ AKT signaling pathway involves several genes listed in Fig. 1B, including PI3K, AKT, CREB5 and PPP2R2C, suggesting that this pathway may play an important role in the development of EMs (Fig. 1C). For further verification, we used qPCR to validate several genes involved in Fig. 1B to confirm the accuracy of RNA-Seq and found that the results were almost consistent with our expectations (Fig. 1D). Of the genes that show progressive changes in the development of EMs, some have been reported and some are weakly associated with the PI3K/AKT signaling pathway 24, 25. After literature review and analysis, the gene we are most interested in is KISS1R.

### 3.2 Abnormal decrease of KISS1R in mice models of EMs

**Figure 2:**
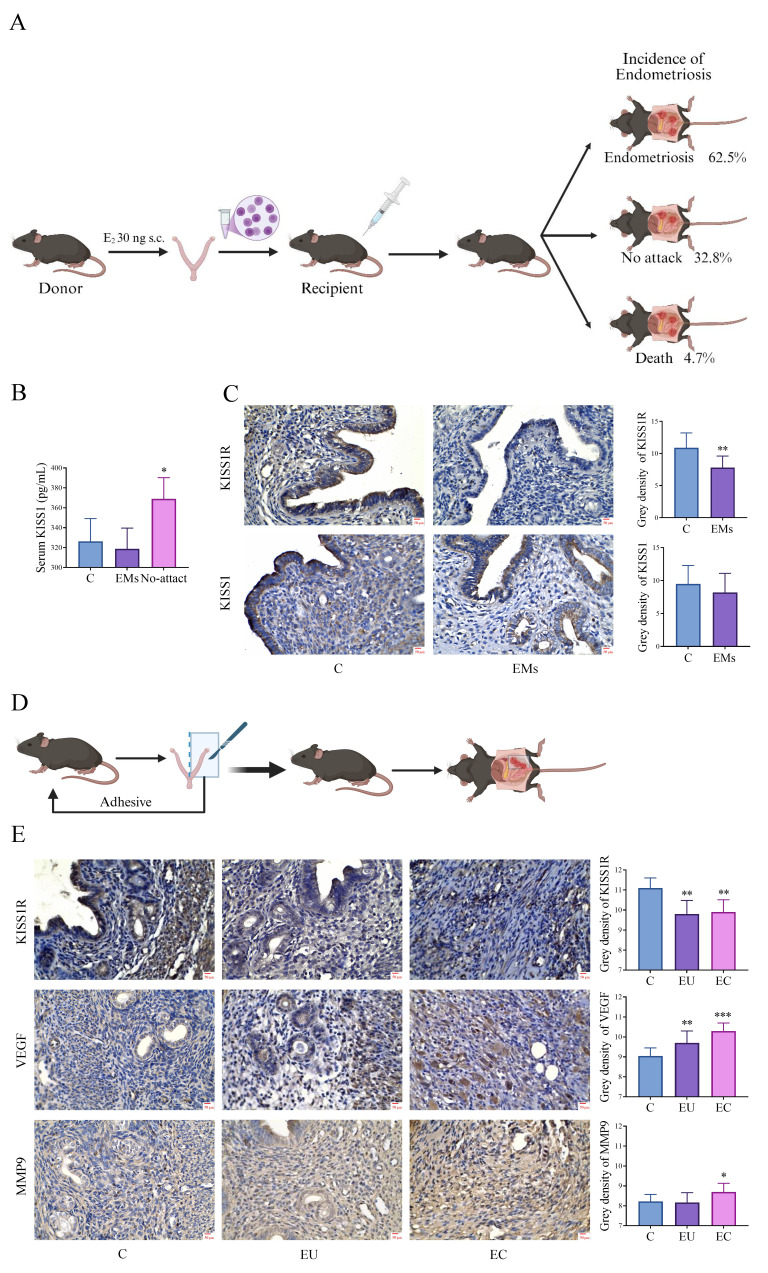
** Abnormal decrease of KISS1R in mice models of endometriosis (EMs). A:** The procedure of the surgical induction in allogeneic mice of EMs. **B:** The level of serum KISS1 in allograft transplantation mice model. C: injected with normal saline; EMs: Model mice with ectopic lesions; No-attack: Model mice without ectopic lesions. **C:** The expression of KISS1R and KISS1 in the recipients' endometrium was detected in the control (C) and EMs groups in the allograft transplantation mouse model. **D:** Flow chart of surgical induction via autologous endometrial transplantation. **E:** KISS1R, VEGF and MMP9 expression in the normal control (C), eutopic endometrium (EU) and ectopic Endometrium (EC) group of the autologous endometrial transplantation mice model. A-B: n = 64 per group. C-E: n = 15 per group. ^*^p <0.05, ^**^p <0.01, ^***^p <0.001 compared with the C group.

After our transcriptomic analysis and validation at the gene level, we focused on KISS1/KISS1R, which may play an important role in the development of EMs. Surgical induction of EMs in allogeneic mice and via autologous endometrial transplantation were conducted to further verify whether KISS1/KISS1R is involved in EMs. In the surgically induced model of allogeneic mice, the incidence of EMs was 62.5% (Fig. 2A). When compared with the non-transplanted group, the serum KISS1 level in the group without EMs after transplantation was significantly increased. However, in mice with EMs after transplantation, the KISS1 levels were markedly low (Fig. 2B).

In addition, according to the immunohistochemical results of control endometrium of non-transplanted control mice and EU in EMs patients, we found differences in the expression of KISS1R and KISS1 in the EU of EMs mice compared to the endometrium of non-transplanted control mice, with a significant decrease in KISS1R and not in KISS1 (Fig. 2C). Subsequently, due to the small size and chaotic organizational structure of EC lesions of the EMs model in allogeneic mice, it is difficult to make morphological quantitative analysis. Therefore, we then used EMs mice model via autologous endometrial transplantation for further investigation. The results showed that compared with the control group, the expression of KISS1R decreased significantly in both EU and EC in EMs mice (Fig. 1E), while VEGF and MMP9 were increased (Fig. 2E). VEGF is the key factor in inducing angiogenesis, and MMP9 plays an important role in cell invasion and degradation of extracellular matrix. The above immunohistochemical results may prove that EMs mice have stronger invasion and angiogenesis ability. Based on the experiment results of EMs mice models, KISS1/KISS1R showed an abnormal decrease in both the allograft model and the autograft model.

### 3.3 Kisspeptins can inhibit the development of EMs

**Figure 3:**
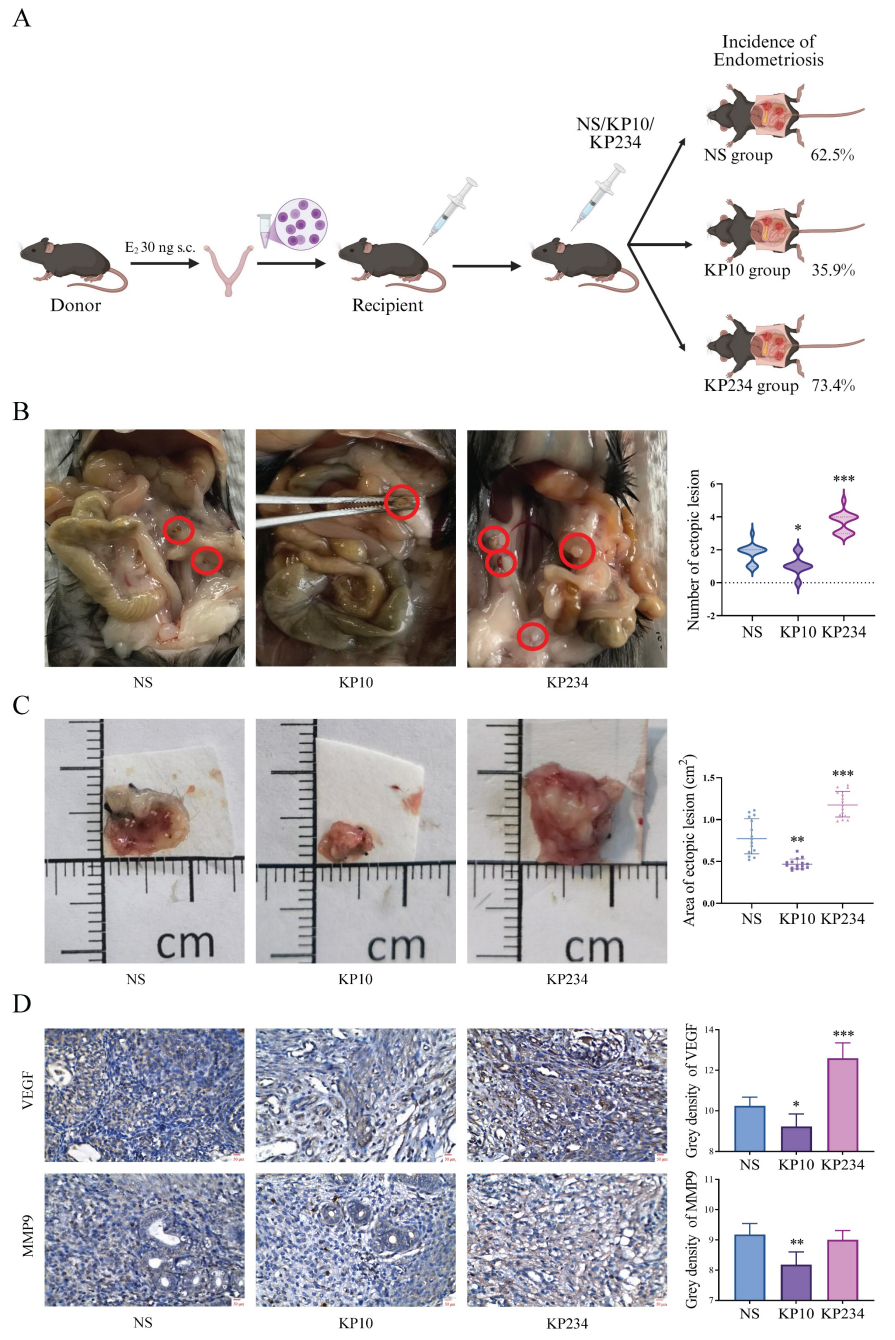
** Kisspeptins can inhibit the development of endometriosis (EMs). A:** The procedure of the surgical induction in allogeneic mice and incidence of EMs among normal saline (NS), kisspeptin-10 (KP10) and kisspeptin-234 (KP234) group of the allograft transplantation mice model. The incidence was 62.5%, 35.4% and 73.4%, respectively. **B:** The ectopic lesion number among NS, KP10 and KP234 group of the allograft transplantation mice model. The ectopic lesion number were 1.80±0.04, 1.00±0.71 and 3.60±0.89, respectively. **C:** The ectopic lesion appearance among NS, KP10 and KP234 group of the autologous endometrial transplantation mice model. The ectopic lesion area was 0.75±0.22, 0.45±0.13 and 1.14±0.18 cm^2^, respectively. **D:** The immunohistochemical results of the expression of VEGF and MMP9 among NS, KP10 and KP234 groups in the autologous endometrial transplantation mice model. A-B: n = 64 per group. C-D: n = 15 per group. ^*^p <0.05, ^**^p <0.01, ^***^p <0.001 compared with the NS group.

To investigate the effect of kisspeptins on EMs, the administration of KP10 was performed during the modeling of EMs. In the allograft model, the incidence was 62.5% in the NS group, which was significantly reduced to 35.4% after treatment with KP10. In contrast, the incidence of EMs in mice treated with KP234 increased to 73.4% (Fig. 3A). Moreover, there were significant differences in the number of ectopic lesions between the NS group and the KP10 group (1.80±0.04 vs 1.00±0.71), as well as between the NS group and the KP234 group (1.80±0.04 vs 3.60±0.89) (Fig. 3B). In the autograft model, the area of the ectopic lesions in the NS group was 0.75±0.22 cm^2^, and significantly reduced to 0.45±0.13 cm^2^ after treatment with KP10. In contrast, the area of the ectopic lesions in mice treated with KP234 increased to 1.14±0.18 cm^2^. According to the immunohistochemical results in the autograft model, the decreased expression of VEGF and MMP9 were observed after the administration of KP10. While KP234 was administered, the opposite results were obtained (Fig. 3D). These results suggest that kisspeptins could possibly inhibit the development of EMs by suppressing invasion and angiogenesis.

### 3.4 Kisspeptins regulate the cell invasion and angiogenesis of EMs

**Figure 4:**
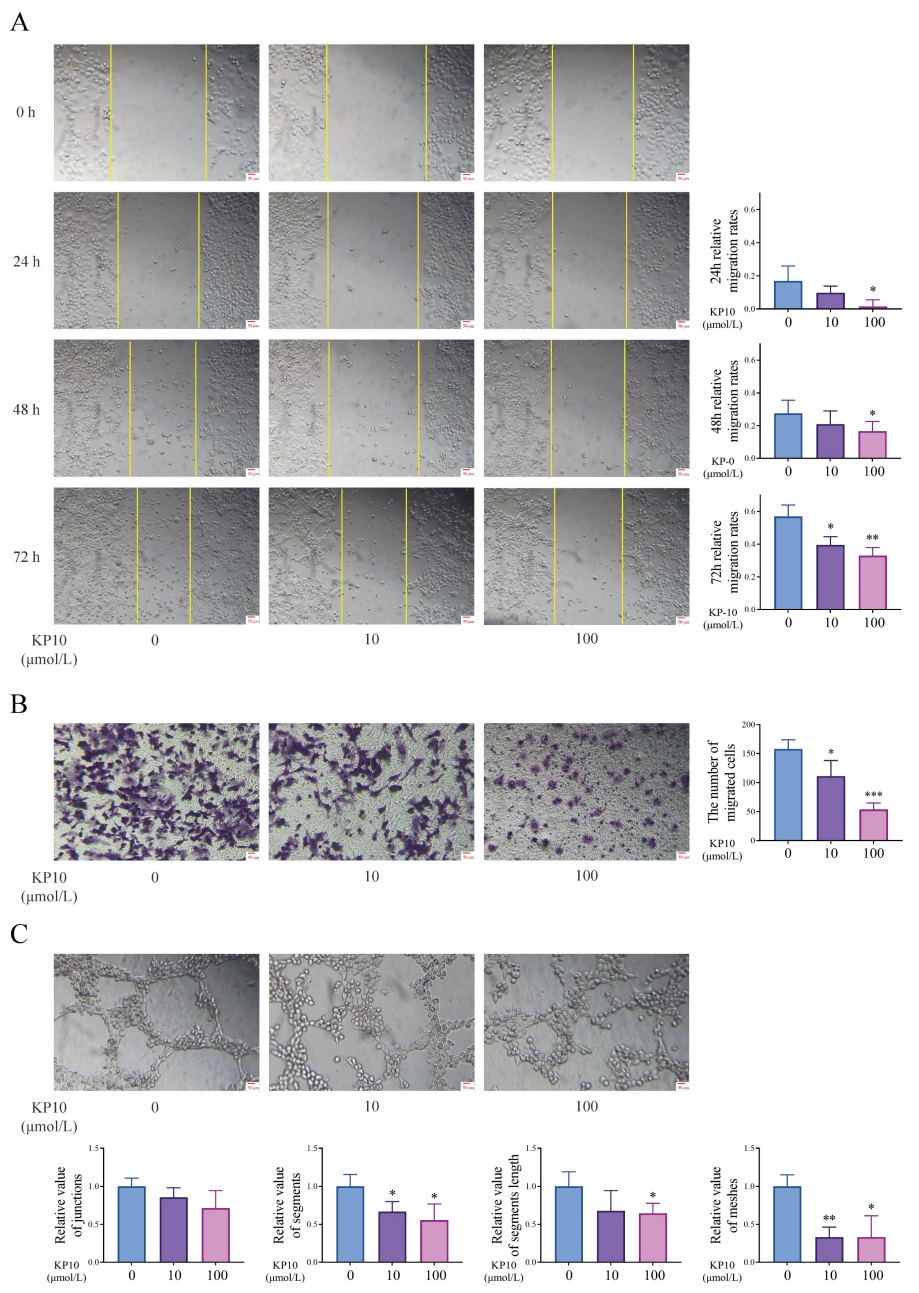
** Kisspeptins regulate the cell invasion and angiogenesis *in vitro* cell experiment. A:** Scratch assay results of human endometrial Ishikawa cells in different concentrations of kisspeptin-10 (KP10) administration conditions. KP10 inhibits Ishikawa cell migration in a dose-dependent manner. **B:** Transwell invasion assay results of Ishikawa in different concentrations of KP10 administration conditions. KP10 inhibits Ishikawa cell invasion in a dose-dependent manner. **C:** Tube formation assay of human umbilical vein endothelial cells (HUVEC) in different concentrations of KP10 administration conditions in 2h. KP10 inhibits angiogenesis in a dose-dependent manner. ^*^p <0.05, ^**^p <0.01, ^***^p <0.001 compared with the none-KP10 group.

**Figure 5:**
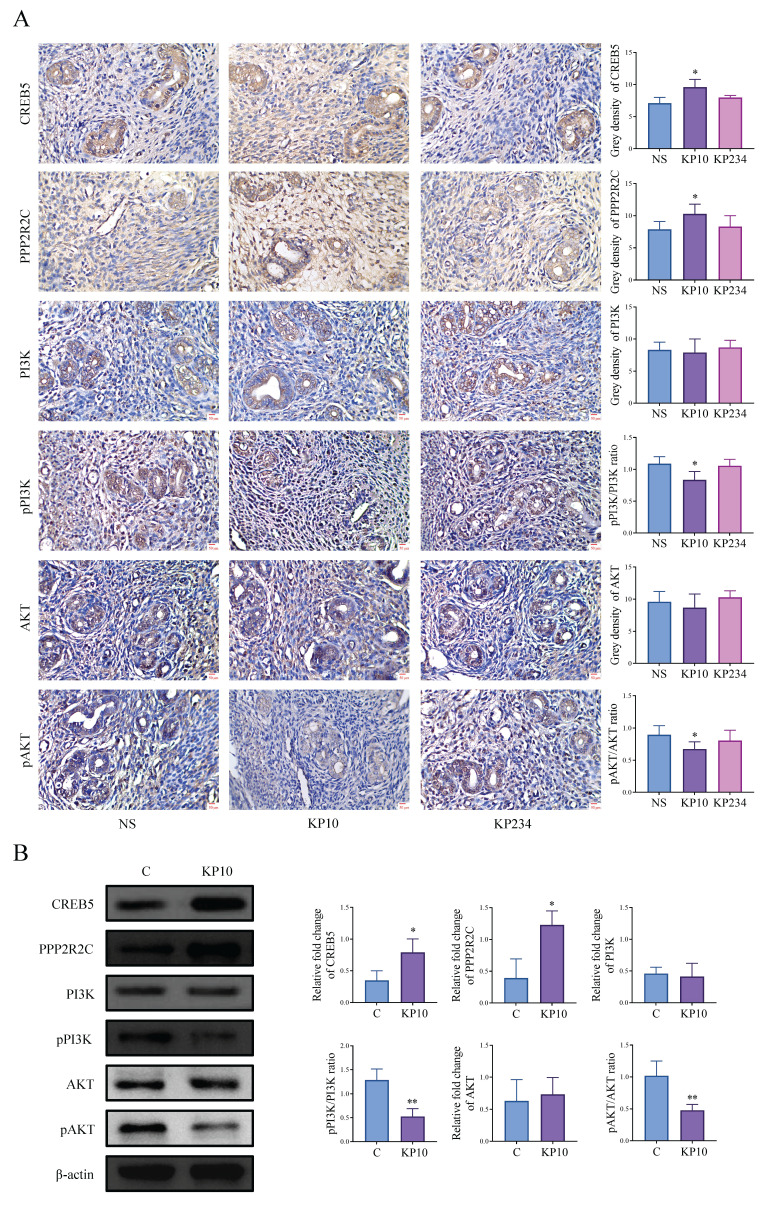
** Kisspeptins modulate the cell invasion and angiogenesis of endometriosis (EMs) through PI3K-AKT signaling pathway. A:** Immunohistochemical results of the key genes associated with PI3K/AKT signaling pathway normal saline (NS), kisspeptin-10 (KP10) and kisspeptin-234 (KP234) group of the autologous endometrial transplantation mice model. n = 15 per group. ^*^p <0.05 compared with the NS group. **B:** Protein bands of PI3K/AKT signaling pathway-related genes in control (C) and KP10 group, as determined by western blot analysis of Ishikawa. KP10 can alter the protein levels of various molecular in the PI3K/AKT signaling pathway. ^*^p <0.05, ^**^p <0.01 compared with the C group.

We conducted cell experiments to analyze the effects of kisspeptins on cell invasion and angiogenesis. Results from the scratch assay and transwell invasion assay showed that exposure to KP10 at concentrations of 0, 10, and 100 µmol/L significantly decreased cell invasion in a dose-dependent manner (Figure 4A-B). In addition, the tube formation assay also displayed that KP10 can inhibit angiogenesis (Fig. 4C). These results align with those of the animal experiments above. What's more, in the autograft model mice, the levels of CREB5 and PPP2R2C in the EC of mice injected with KP10 were significantly higher than those in the control group injected with NS, whereas the ratios of pPI3K/PI3K and pAKT/AKT were significantly reduced (Fig. 5A). According to the Western blot experiment results of Ishikawa, when compared with controls, we found that after KP10 administration, the expressions of CREB5 and PPP2R2C increased, while pPI3K/PI3K and pAKT/AKT ratios decreased (Fig. 5B).

### 3.5 Kisspeptins regulate the EMs through PI3K/AKT signaling pathway via CREB5

**Figure 6:**
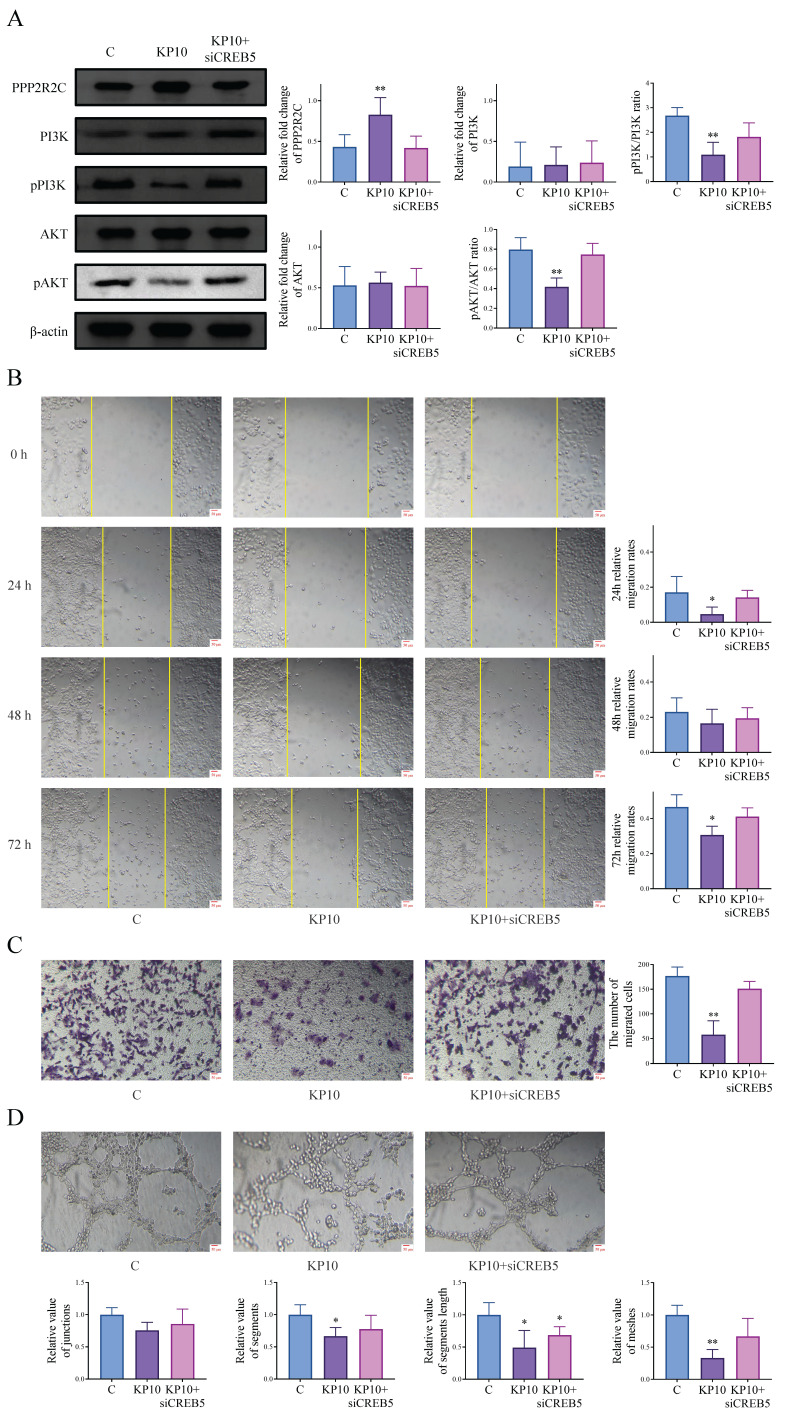
** Kisspeptins regulate the endometriosis (EMs) through PI3K-AKT signaling pathway via CREB5. A:** The protein levels of PI3K/AKT signaling pathway-related genes PPP2R2C, PI3K, pPI3K, AKT, pAKT, in control (C), kisspeptin-10 (KP10) and KP10+siCREB5 group in Ishikawa, as determined by western blot analysis. KP10 can alter the protein levels of various molecular in the PI3K/AKT signaling pathway through CREB5. **B:** Scratch assay results of Ishikawa among C, KP10 and KP10+siCREB5 group. KP10 inhibits Ishikawa cell migration through CREB5. **C:** Transwell invasion assay results of Ishikawa among C, KP10 and KP10+siCREB5 group. KP10 inhibits Ishikawa cell invasion through CREB5. **D:** Tube formation assay of HUVEC among C, KP10 and KP10+siCREB5 group in 2h. KP10 inhibits angiogenesis through CREB5. ^*^p <0.05, ^**^p <0.01 compared with the C group.

According to transcriptome results, CREB5 is also differentially expressed in the PI3K/AKT pathway, which has also been verified in the above animal and cell experiments. As CREB5 is well known as a recognized transcription factor, we speculated that kisspeptins might modulate EMs through CREB5 and conducted a series of experiments. After the cells were treated with KP10, PPP2R2C was upregulated, while the ratios of pPI3K/PI3K and pAKT/AKT were decreased. When cells were treated with KP10 and CREB5 was knocked down, the corresponding protein expression levels changed in reverse (Fig. 6A). We also conducted cell experiments for further verification. The scratch assay and transwell invasion assay showed significant inhibition of cell invasion after KP10 administration compared to the control group, but inhibition was reversed after KP10 intervention and CREB5 knockdown (Fig. 6B-C). Based on the tube formation assay, we observed that KP10 inhibited angiogenesis, and the inhibition was reversed after the intervention of KP10 and the knockdown of CREB5 (Fig. 6D). These results suggest that kisspeptins may inhibit EMs by regulating the PI3K/AKT signaling pathway through CREB5 via PPP2R2C.

## 4. Discussion

According to the generally recognized pathogenesis of EMs, the theory of retrograde menstruation, the EU is also the pathological endometrium, whereas the EC is the pathological tissue where the lesion is aggravated and which is more similar to the biological characteristics of tumor cells. However, the specific process by which this transformation occurs may involve a series of complex and progressive changes. This indicates that the key to the pathogenesis of EMs may be the EU itself. In other words, it is of great theoretical value to search for the early molecular characteristics of EMs and explain its internal mechanism. Based on the transcriptome analysis in this study, we found some DEGs, among which the molecules that conform to this progressive change are mainly enriched in PI3K/AKT signaling pathway, phospholipase D signaling pathway and cGMP-PKG signaling pathway. In accordance with the reports of relevant studies, although the phospholipase D signaling pathway and cGMP-PKG signaling pathway are related to molecules with progressive changes to a certain extent, PI3K/AKT involves more molecules with progressive changes and has more literature support. An Italian clinical study demonstrated that the expression of phospholipase D was detected in EMs, albeit at levels comparable to those in controls 26. In addition, a bioinformatics study concerning infertile EMs indicated that the intersection genes of EMs and infertility are predominantly concentrated in the metabolism of different amino acid and cGMP-PKG signaling pathway 27. Moreover, several researchers have discovered that the PI3K/AKT signaling pathway gets activated in the context of EMs, with the levels of pyroptosis and inflammatory factors being remarkably elevated 28. Additionally, an Argentine clinical study revealed that there was an elevated expression of PI3K and enhanced AKT phosphorylation levels in both the EU and EC of patients suffering from EMs in contrast to the control endometrium 18. Besides, AKT is highly phosphorylated in ovarian EMs compared to controls without EMs 29. These studies all imply that PI3K/AKT is of great significance in EMs and exhibits a high level of expression.

Among the DEGs with progressive changes, we focused on KISS1R. Nevertheless, research on the pathways associated with KISS1 is scarce and its function within the context of EMs remains unclear. There are several clinical studies concerning KISS1 in EMs, yet little has been reported about KISS1R. The recent research on EMs demonstrated that the expression of KISS1 was lower in patients with EMs than in controls via immunofluorescence assay 7. In addition, it has been demonstrated by prior studies that the expression of KISS1 is remarkably augmented in the EC of EMs patients when compared with the controls free of EMs 30. Furthermore, previous studies have shown that KISS1 and KISS1R expression levels are significantly reduced in both EU and EC of women with EMs 31. We have been concerned about KISS1/KISS1R for a long time. Based on existing clinical studies, we conducted a systematic review and found that KISS1 has a tendency of low expression in EMs in accordance with the Supplementary File, but KISS1R is rarely reported. In light of the aforementioned analysis, we initiated animal experiments and proved that KISS1 exhibited diminished expression in both the allograft model and the autograft model mice of EMs. Besides, we also found that the no-attack group exhibited a significantly higher serum KISS1 level compared to the EMs group and the control group, which suggests that KISS1 may act as a protective factor against endometrial ectopic implantation. According to the theory of retrograde menstruation, it is common for endometrial tissue to flow into the pelvic cavity. However, most women do not develop endometriosis, indicating that ectopic implantation does not typically occur. This suggests that endometriosis may only arise if the endometrial tissue itself is altered or if the inherent protective mechanisms fail. Our results provide further evidence for the above hypothesis, with KISS1 being a key protective factor. In addition, further functional verification was conducted, and it was clearly confirmed that KISS1 is capable of suppressing the occurrence of EMs. Our results demonstrated that KISS1 led to a reduction in the incidence of the allograft model mice, inhibited the growth of lesions in the autograft model mice.

Research on the physiological and pathological functions of KISS1 in the endometrium has made significant progress. The research indicated that kisspeptins were found at elevated levels across the endometrium on the fourth day of pregnancy in mice, implying that kisspeptins might have a function in the modulation of endometrial receptivity 32. It modulates the hormonal environment and cell signaling pathways that are crucial for the proper implantation of the embryo. In pathological aspects, it has been proved that KISS1 can suppress cell invasion and angiogenesis in multiple types of tumors as well as in germ cells. Previous research has revealed that KISS1 can not only trigger the proliferation of prostate cancer but also contribute to the metastasis by augmenting the migration, invasion, and angiogenesis of malignant cells 33. Additionally, former investigations have demonstrated that KISS1 restrains the angiogenesis of breast cancer brain metastases 34. Furthermore, kisspeptins have been shown to suppress the cell invasion and angiogenesis of human trophoblast cells during the early stage of pregnancy 35. Similar to malignant tumors, one of the characteristics of EMs is abnormal cell invasion and angiogenesis. To determine whether KISS1 inhibits cell invasion and angiogenesis through KISS1R, we performed scratch wound healing assay, cell invasion assay, and tube formation assay. These experiments showed that KP10 significantly inhibit the migration and invasion of Ishikawa and the angiogenesis of HUVEC, which suggest that KISS1 may suppress cell invasion and angiogenesis, thereby impeding the occurrence and development of EMs. The precise molecular mechanisms underlying these pathological alterations remain incompletely explained. Additional research is required to comprehensively elucidate the function of KISS1 in EMs.

Evidences have shown that the PI3K/AKT pathway modulates the proliferation, adhesion, migration, angiogenesis and invasion of endometrial cells in the context of EMs 36-38. In the treatment of EMs, Tanshinone IIA plays a role by means of cell invasion, angiogenesis and suppressing PI3K/AKT signaling pathways 39. In addition, Flavokawain A exerts a beneficial impact on a rat model of surgically induced EMs by means of modulating the PI3K/AKT signaling pathway to reduce angiogenesis 40. What's more, in existing clinical studies, it has turned out that the PI3K/AKT signaling pathway shows hyperexpression in the case of EMs 6, 41. In addition, it has been demonstrated by previous investigations that the PI3K/AKT signaling pathway is likely to enhance cell invasion and angiogenesis via mTOR, eNOS and VEGF 42-45. According to previous literature, Integrins, FGFR, and GPCRs are known to activate the PI3K/AKT signaling cascade 46-48. Conversely, it was reported that the PI3K/AKT signaling pathway could be inhibited by RTKs, EGFR and KISS1 49-51. In our research, through animal and cell experiments, it was discovered that KISS1 suppresses the PI3K/AKT signaling pathway. However, the molecular mechanisms that contribute to the changes in biological behavior regarding angiogenesis and cell invasion in EMs remain incompletely understood. Through our study and based on the sequencing results, it was found that CREB5 is also a gene with differential expression. Furthermore, numerous studies have indicated that CREB5, functioning as a transcription factor, is capable of regulating the PI3K/AKT signaling pathway 20, 52 . Moreover, in line with the experiment results, we found that KP10 intervention inhibited cell invasion and angiogenesis in EMs by modulating the PI3K/AKT signaling pathway in both allograft and autograft models. Based on cell experiments, the changes in pPI3K/PI3K and pAKT/AKT expression detected by Western blot showed that KP10 intervention reduced the ratios of pPI3K/PI3K and pAKT/AKT, and these results were reversed after CREB5 knockdown. In conclusion, KISS1 regulates cell invasion and angiogenesis in EMs through the CREB5-mediated PI3K/AKT signaling pathway.

A large number of studies have been document concerning the molecular mechanism through which KISS1 modulates cell invasion and angiogenesis. It is held by certain scholars that KP10 restrains tumor angiogenesis via the suppression of Sp1-mediated VEGF expression as well as FAK/Rho GTPase activation 53. Additionally, research findings indicate that when KISS1 is overexpressed, it can lessen the invasion of colorectal cancer cells by impeding the PI3K/AKT signaling pathway 54. According to these literatures, the existing research regarding the molecular mechanism through which KISS1 functions is limited and has not involved whether KISS1 has the effect of promoting gene transcription. In our study, we have identified a novel molecular mechanism of KISS1, which has not been documented in other cells and tissues. Furthermore, the results suggest that KISS1 may exert its effects by influencing CREB5 expression. A series of experiments were carried out to verify this functional mechanism.

In fact, there are some limitations in our study. First, the principal defect of the present study lies in its limited sample size. Subsequent studies ought to strive to surmount the constraints of this research by enlarging the sample size. Second, due to ethical constraints in specimen collection, we were unable to recruit completely healthy women as the normal control group. Instead, we used women without EMs but with other benign gynecological conditions as controls. In addition, this study merely represents a preliminary exploration of the molecular mechanisms that promote CREB5 through KISS1. Future studies ought to delve into more detailed molecular mechanisms.

Overall, our data reveal that KISS1 inhibits cell invasion and angiogenesis in EMs, and this gene exerts its function by reducing CREB5 expression via blocking of the PI3K/AKT pathway. The therapeutic effect of KISS1 is not only reflected in the reduction of lesions, but also inhibits the development of EMs. The elucidation of signal transduction mediators participating in this process may promote the development of specific inhibitors for modulating cell invasion and angiogenesis in EMs clinically.

## 5. Conclusion

In our study, the involvement of KISS1/KISS1R was identified in the transcriptome results. In addition, we have verified the function of KISS1/KISS1R in EMs through animal and cell experiments, which may play an important role through the PI3K/AKT signaling pathway via CREB5. This finding may offer additional explanations for the pathogenesis of EMs and suggest targeted therapeutic strategies for EMs patients.

## Supplementary Material

Supplementary figure and table.

## Figures and Tables

**Table 1:** Primer sequences used for quantitative real-time PCR.

Genes	Primer sequence (5'-3')
GAPDH-F	AACATCATCCCTGCCTCTACTGG
GAPDH-R	CCTCCGACGCCTGCTTCAC
PPP2R2C-F	CACCTACCACATCAACTCCATCT
PPP2R2C-R	GCTTGATGTCCACGATGTTGAAG
FN1-F	GAGAATAAGCTGTACCATCGCAA
FN1-R	CGACCACATAGGAAGTCCCAG
MMP9-F	TGACAGCGACAAGAAGTG
MMP9-R	CAGTGAAGCGGTACATAGG
PI3K-F	TGCCAAACCACCTCCCATTCCT
PI3K-R	CATCTCGTTGCCGTGGAAAAGC
VEGF-F	TCCTCACACCATTGAAACCA
VEGF-R	GATCCTGCCCTGTCTCTCTG
CREB5-F	CTGGAGCGGGCTACTTGTATATT
CREB5-R	CCTCTCCTGCTCCAAATTCATCT
AKT-F	CAGGAGGAGGAGGAGATGGACTTC
AKT-R	AGGTACTCAAACTCGTTCATGGTCAC
WNT10A-F	TCGCAACAAGATCCCCTATG
WNT10A-R	GCAGTGCATCCAGTTGTAAG
KISS1R-F	GGACCGTGACCAACTTCTACAT
KISS1R-R	GTTGACGAACTTGCACATGAAG

**Table 2:** Demographic characteristics of control and EMs groups

	Age	BMI	Age at menarche	Height	Weight	rAFS score/stage	Type of benign condition	Location of the tissue/ pathological diagnosis
C1	30	21.88	12	1.60	56	-	Endometrial polyps	Uterine: endometrium (proliferative phase)
C2	33	20.78	13	1.52	48	-	Uterine fibroids	Uterine: endometrium (proliferative phase)
C3	28	19.92	13	1.60	51	-	Endometrial polyps	Uterine: endometrium (proliferative phase)
EMs1	37	18	13	1.65	49	27/Ⅲ	-	Uterine: endometrium (proliferative phase)
								Ovary: endometrium
EMs2	39	20.34	13	1.56	49.5	41/Ⅳ	-	Uterine: endometrium (proliferative phase)
								Ovary: endometrium
EMs3	28	19.56	13	1.55	47	37/Ⅲ	-	Uterine: endometrium (proliferative phase)
								Ovary: endometrium
EMs4	30	18.82	13	1.63	50	40/Ⅲ	-	Uterine: endometrium (proliferative phase)
								Ovary: endometrium
EMs5	26	24.46	14	1.62	52	44/Ⅳ	-	Uterine: endometrium (proliferative phase)
								Ovary: endometrium
