# Supplementary material for: Kisspeptins inhibit ectopic endometrial cell invasion and angiogenesis by suppressing PI3K/AKT signaling pathway via CREB5 in endometriosis
Source: Int J Med Sci. 2026 Jan 1;23(1):334–49. doi: 10.7150/ijms.112890 (PMC12702097; doi:10.7150/ijms.112890)
Supplement: Supplementary file 1 — Supplementary figure and table. [file ijmsv23p0334s1.pdf]

Supplementary Figure 1. Forrest plot for the effect of KISS1 positive expression ratio in EMs patients and controls

Study or Subgroup	Ectopic and Ectopic Events	Ectopic and Ectopic Total	Control Events	Control Total	Weight	Odds Ratio M-H, Fixed, 95% CI
Fu 2012	39	78	14	27	5.7%	0.93 [0.39, 2.23]
Jin 2013	21	40	15	20	5.2%	0.37 [0.11, 1.21]
Kolioulis 2017	55	58	24	29	0.9%	3.82 [0.84, 17.28]
Li 2015	89	256	85	114	41.7%	0.18 [0.11, 0.30]
Liu 2009	26	70	23	35	10.5%	0.31 [0.13, 0.72]
Makri 2012	3	55	0	16	0.4%	2.20 [0.11, 44.83]
Peng 2008	14	46	35	55	12.1%	0.25 [0.11, 0.58]
Shen 2007	19	62	21	33	10.3%	0.25 [0.10, 0.62]
Shi 2012	26	80	25	40	12.2%	0.29 [0.13, 0.64]
Tatiana 2016	14	19	5	8	1.0%	1.68 [0.29, 9.75]
**Total (95% CI)**		**764**		**377**	**100.0%**	**0.33 [0.25, 0.44]**
Total events	306		247			

Heterogeneity: Chi² = 26.83, df = 9 (P = 0.001); I² = 66%
Test for overall effect: Z = 7.95 (P < 0.00001)

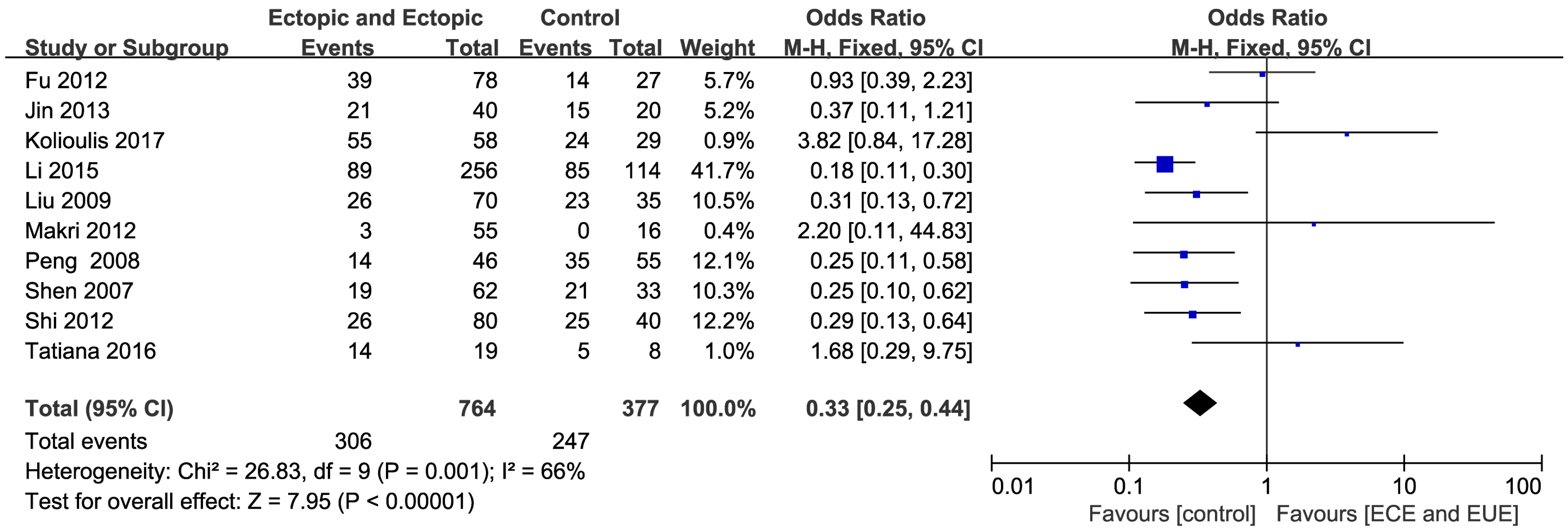

Data unpublished.

Supplementary Table 1. Main characteristics of the included studies

Author	Year	Country	Study design	Age(case/ control)	Number of patients		Ectopic position	Endometriosis grade	Definition of the control group	Laboratory method	Outcome measures
					Case	Control					
Makri[1]	2012	Greece	Case-control study	30.1 (22-41)/31.6(18-44)	24	16	ovarian and infiltrating	III - IV	patients without endometriosis undergoing laparoscopy for other benign gynecological reasons (ovarian cystic lesions, uterine fibromas, diagnostic laparoscopy for chronic pelvic pain)	RT-PCR and IHC	KiSS-1 and KiSS-1R
Fu[2]	2012	China	Case-control study	43.65±4.21 (27-48)/46.35 ±3.68	48	30	ovarian	III - IV	patients without endometriosis undergoing laparoscopy or open hysterectomy	IHC	KiSS-1 and MMP-9
Kolioulis [3]	2017	Greece	Case-control study	48 (36-67)/34(17-45)	29	29	myometrium	-	patients without adenomyosis, but with other benign disease (ovarian mature cystic teratoma luteal cysts, follicular cysts, hydrosalpinges, ovarian serousand mucinous cystadenomas, uterine fibroids, bothuterine fibroids and ovarian serous cystadenoma and unexplained infertility)	IHC	KiSS-1
Jin[4]	2013	China	Case-control study	37.5 (24-53)	40	20	myometrium	-	patients with adenomyosis	IHC	KiSS-1 and MMP-9
Li[5]	2015	China	Case-control study	36±9 (14-46)/37±10 (14-50)	256	114	-	I - IV	patients without endometriosis, but with other benign gynecological disease (benign ovarian tumor, mesosalpinx cyst and cervical lesion)	IHC	KiSS-1、sflt-1、VEGF、MCP-1 and RANTES
Liu[6]	2009	China	Case-control study	45.26/47.06	93	105	-	I - IV	patients without endometriosis, undergoing curettage before hysterectomy or myomectomy for subserous or intramural uterine fibroids	IHC	KiSS-1 and OPN
Peng[7]	2008	China	Case-control study	34.87/40.06	46	55	ovarian	-	patients without endometriosis, undergoing curettage before hysterectomy or myomectomy for subserous or intramural uterine fibroids	IHC	KiSS-1
Shen[8]	2007	China	Case-control study	34.87,41.06/40.06	46	33	ovarian	I - IV	patients without endometriosis, undergoing curettage before hysterectomy or myomectomy for subserous or intramural uterine fibroids	IHC	KiSS-1 and MMP-9
Shi[9]	2012	China	Case-control study	-	40	40	ovarian	I - IV	patients with uterine fibroids undergoing hysterectomy	IHC	KiSS-1
Tatiana [10]	2016	Russia	Case-control study	32.3/31.5	10	8	-	II, III	healthy women	IHC	KiSS-1 and KiSS-1R

Data unpublished.

The forest plot was based on immunohistochemical results from 10 case–control studies (590 cases and 377 controls). Therefore, three studies using immunofluorescence and ELISA were not included. [11-13]

Reference

1. Makri, A., et al., *KISS1/KISS1R expression in eutopic and ectopic endometrium of women suffering from endometriosis.* In Vivo, 2012. **26**(1): p. 119-27.
2. Xianghong, F., X. Jianmei, and W. Ruijin, *Research on Expression of KISS-1/MMP-9 in Ectopic and Eutopic Endometrium from Women with Endometriosis and Its Biological Significances.* Asia-Pacific Traditional Medicine, 2012. **8**(12): p. 5-7.
3. Kolioulis, I., et al., *Immunohistochemical expression pattern of metastasis suppressor KISS-1 protein in adenomyosis lesions and normal endometrium.* Eur J Obstet Gynecol Reprod Biol, 2017. **210**: p. 64-68.
4. Haihong, J., et al., *Expression and Clinical Significance of KISS-1、MMP-9 in Adenomyosis.* Chin J Lab Diagn, 2013. **17**(8): p. 1446-1448.
5. Li, P., et al., *The expression of sflt-1, VEGF, MCP-1, RANTES and kiss-1 in serum and peritoneal fluid of endometriosis patients with metabolic syndrome.* Hebei Medicine, 2015. **37**(15): p. 2276-2278.
6. Zhou, L., et al., *Expression and Significance of Kiss-1 and Osteopontin in Ectopic and Eutopic Endometrium in Patients with Endometriosis.* Chin J Clin Obstet Gynecol, 2009. **10**(6): p. 442-444.
7. Laiqin, P., et al., *Expression and Significance of KISS-1 in Ectopic Endometrium in Patients with Endometriosis.* Chin J Prim Med Pharm, 2008. **15**(6): p. 905-906.
8. Qingwen, S., et al., *Expression and Significance of KISS-1 and Matrix Metalloproteinase-9 in Ectopic and Eutopic Endometrium in Patients with Endometriosis.* Prog Obstet Gynecol, 2007. **16**(11): p. 843-845.
9. Shi, J. and H. Wang, *Expression and significance of KISS - 1 in endometriosis.* Inner Mongolia Traditional Chinese Medicine, 2012. **31**(06): p. 78-79.
10. Kleimenova, T., et al., *Identification of kisspeptins in endometrial cell culture from patients with endometriosis and ex vivo.* Gynecological Endocrinology, 2016. **32**: p. 88-88.
11. Akad, M., et al., *Kisspeptin Serum Levels in Patients with Endometriosis, New Research Pathways Regarding Female Infertility.* Maedica (Bucur), 2022. **17**(3): p. 557-560.
12. Onal, M., et al., *Serum kisspeptin levels in deep-infiltrating, ovarian, and superficial endometriosis: A prospective observational study.* Medicine (Baltimore), 2022. **101**(45): p. e31529.
13. Kleimenova, T., et al., *The Expression of Kisspeptins and Matrix Metalloproteinases in Extragenital Endometriosis.* Biomedicines, 2024. **12**(1).
